# Semaphorin-3E Produced by Immature Dendritic Cells Regulates Activated Natural Killer Cells Migration

**DOI:** 10.3389/fimmu.2018.01005

**Published:** 2018-05-09

**Authors:** Abdulaziz Alamri, Rahmat Rahman, Manli Zhang, Abeer Alamri, Abdelilah S. Gounni, Sam K. P. Kung

**Affiliations:** ^1^Department of Immunology, University of Manitoba, Winnipeg, MB, Canada; ^2^Department of Oral Biology, University of Manitoba, Winnipeg, MB, Canada

**Keywords:** semaphorins, innate immunity, cell trafficking, cytokines activation, TLR ligands, natural killer cells, dendritic cells

## Abstract

Natural killer (NK) cells and dendritic cells (DCs) are two innate immune cells that are critical in regulating innate and adaptive immunity. Cellular functions and migratory responses of NK or DC can be further regulated in NK-DC crosstalk that involves multiple cytokine signals and/or direct cell-cell contacts. Semaphorin-3E (Sema-3E) is a member of a large family of Semaphorin proteins that play diverse regulatory functions in different biological systems upon its binding to the cognate receptors. However, possible role(s) of Sema-3E on the regulation of NK-cell functions has not been elucidated. Here, we first demonstrated that DC and NK cells expressed Sema-3E and its receptors, respectively. To formally address the importance of DC-derived Sema-3E in regulating NK-cell migration, we compared *in vitro* migratory responses of activated NK cells (aNKs) toward different conditioned media of DCs (immature, lipopolysaccharide- or Poly I:C-stimulated) derived from Sema-3E^+/+^ or Sema-3E^−/−^ mice. We observed that aNKs exhibited enhanced migrations toward the conditioned medium of the immature Sema-3E^−/−^ DC, when compared with that of the immature Sema-3E^+/+^ DC. Addition of exogenous recombinant Sema-3E to the conditioned medium of the Sema-3E^−/−^ immature DC (iDC) abrogated such enhanced NK-cell migration. Our current work revealed a novel role of Sema-3E in limiting NK-cell migrations toward iDC in NK-DC crosstalk.

## Introduction

Natural killer (NK) cells are members of the emerging family of innate lymphoid cells that play important roles in innate immunity and tissue remodeling (1, 2). They migrate to peripheral or inflamed tissues to exert their immune-surveillance functions (3). They are equipped with competent cytolytic machinery that do not require prior sensitization to recognize and eliminate abnormal cells (4). Upon activations, NK cells are also capable of producing a number of different cytokines/chemokines (such as IFN-γ, TNF-α, GM-CSF, MIP-1α, MIP-1β, CCL5, and RANTES) that either activate or help recruit other cell types to the tissues to shape anti-tumor or anti-microbial responses (5–8).

Individual NK cell expresses a repertoire of activating and inhibitory receptors on the cell surface that, upon engagements of their cognate ligands, will determine the outcome of NK-cell activities (2, 4, 5, 8). In addition, NK cells can be activated by an array of cytokines (such as IL-2, IL-12, IL-15, and IL-18) (9–12), or dendritic cell (DC) in the microenvironment (13–15). DCs are professional antigen-presenting cells that engage T cell receptor with the cognate MHC/Peptide complex (Signal 1) and co-stimulatory molecules (Signal 2) to regulate optimal T cell activation. Depending on their maturation and activation states, DC acquires specific ability to induce either immunological tolerance or differentiation of distinct T cell subsets (14, 16). Interactions of NK and DC are bi-directional, involve multiple cytokine signals and/or direct cell–cell contacts (17). Regulation of DC maturation/functions by NK cells presents another indirect mechanism for NK cells to coordinate innate and adaptive immune responses *in vivo* (18–20).

Semaphorins were first reported as axon-guidance molecules in the nervous system (21). Subsequent studies revealed a large family of secreted and membrane-bound semaphorin members that regulate multiple cellular systems (such as nervous, immune, respiratory, and cardiovascular systems), physiological processes (such as angiogenesis and embryogenesis), as well as pathological conditions (such as airway diseases and tumor formation) (21). Most semaphorin molecules mediate their functions by direct and selective binding of their cognate plexins and neuropilins (NRPs) receptor that can exist either as homomeric or heteromeric complexes (22–24). Semaphorin-3E (Sema-3E) was originally identified as an axon-guidance cue in neural development. However, its wide expression in non-neural cell types and the dys-regulation of Sema-3E expression in cancers, autoimmunity, and allergic diseases suggested their diverse regulatory roles in multiple systems (25). Binding of Sema-3E to the Plexin D1 receptor was of high affinity, and can be independent of the NRP co-receptor (26). Such interaction activated the intracellular Plexin D1 RasGAP (Ras GTPase activating protein) domain and reduced R-Ras activity (27).

Our recently published work in allergic inflammatory and asthma models reported a regulatory role of Sema-3E in the development and maintenance of allergic asthma (28, 29). Holl et al. reported that Plexin D1-deficient DC produced selectively higher level of IL-12/IL-23 p40 (29). Collectively, they further established a critical role of Sema-3E in the modulation of immune responses (30). Here, we examined formally whether Sema-3E exerts any regulatory function on NK cells in NK-DC crosstalk. We first examined the expression of Sema-3E and its receptors on NK and DCs. We also examined whether Sema-3E regulated aNK migration in NK-DC crosstalk.

## Materials and Methods

### Animals and Ethics Statement

Sema-3E^+/+^ or Sema-3E^−/−^ BALB/c mice were maintained and housed at University of Manitoba, Winnipeg, Canada. All mice were maintained in Animal Care facility, the University of Manitoba under pathogen-free conditions, and used according to the guidelines specified by the Canadian Council for Animal Care. Parent breeders of these animals were gifted by Dr. F. Mann, Université de la Méditerranée, Marseille, France. Research ethics boards of the University of Manitoba, Winnipeg, Canada, approved the current study (protocol # 13-018).

### Antibodies and Flow Cytometry

Antibodies used in this study are DX5 (DX5), CD3 (145-2C11), CD40 (1C10), CD86 (GL1), CD80 (16–10A1), from eBiosciences (San Diego, CA, USA), and from BD Pharmingen (NJ, USA). Anti-human/mouse Sema-3E, anti-human Plexin D1 (85% cross- reactivity with mouse) (30), and mouse NRP1 antibodies were purchased from R&D system (Minneapolis, MN, USA). NK or DC was incubated with anti-Fcγ RIII (2.4 G2) before surface marker staining. In surface staining, NK and DC cells were incubated with Fc-blocker (eBiosciences) in flow tubes for 10 min on ice. The cells were then incubated with the specified antibodies in flow buffer (PBS supplemented with 2% FBS) for 20 min at 4°C. NK cells were stained with anti-DX5, CD3 mAbs (at 10 µg/ml) (eBiosciences) and/or Sema-3E, Plexin D1, and NRP1 fluorochrome-conjugated monoclononal antibodies (at 10 µg/ml) (R&D). DCs were stained with anti-CD11c, CD40, CD86, CD80 (eBiosciences) monoclonal antibody and/or anti-Sema-3E, Plexin D1, and NRP1 fluorochrome-conjugated monoclononal antibodies (R&D) on ice. Cells were washed in the flow buffer, fixed with 2% para-formaldehyde (PFA) before flow cytometric analyses. For intracellular staining, cells were fixed with 4% PFA, permeabilized with 0.1% saponin (Sigma-Aldrich) in flow buffer and then stained with Sema-3E, Plexin D1, or NRP1 fluorochrome-conjugated monoclononal antibodies (R&D) for 30 min on ice. Surface and intracellular stained samples acquisition was performed on an FACSCanto II (BD Biosciences) using Diva software and data were analyzed using FlowJo software.

### Recombinant Proteins

Recombinant human Plexin D1 and recombinant mouse Sema-3E were purchased from R&D system (Minneapolis, MN, USA). Recombinant mouse CXCL12, recombinant mouse CXCL10, and recombinant mouse CCL19 were purchased from eBiosciences (San Diego, CA, USA).

### Preparation of DCs Conditioned Medium

Bone-marrow cells were stimulated to generate mature DCs (31). Precursor cells were extracted from the femur and tibia and incubated with ACK buffer for 2 min to lyse red blood cells. 0.5–1 × 10^6^ BM cells per well were seeded in a 24-well plates containing RPMI 1640 (Hyclone) medium supplemented with 1% PSG, 10% FGS, 1.6 mmol/l 2-mercaptoethanol (2-ME), and 20 ng/ml GM-CSF (Peprotech). On day 3, one-third of the culture medium was aspirated to remove non-adherent cells and supplemented with fresh GM-CSF containing medium. On day 5, cultures were replenished with fresh GM-CSF medium while maintaining total volume 1 ml/well. On day 8, lipopolysaccharide [LPS—1 µg/µl and Polyinosinic:polycytidylic acid 10 µg/µl (Poly I:C from Sigma)] both were introduced in the culture for 24 h to acquire matured DC phenotype. DCs with or without LPS or Poly I:C treatment and the corresponding culture conditioned media were used in various combinations and settings throughout these experiments. The expression of CD40, CD80, and CD86 surface markers represent mature DC phenotype. All mature and immature DC (iDC) cells were confirmed by flow cytometery before each experiment (Figure S1 in Supplementary Material).

### Quantitative Real-Time PCR

We used cell sorting to isolate NK cells for qPCR analyses. In brief, single-cell suspension of splenocytes was obtained from BALB/c mice. We first used EasySep mouse CD19 positive selection kit (StemCell Technologies, Vancouver, BC, USA) according to the manufacturer’s protocol to remove B cells from the splenocyte preparations. The NK-enriched splenocyte preparations were subjected to FACSorting for CD3^−^DX5^+^ NK cells using anti-DX5 and anti-CD3^−^ antibodies.

The sorted NK cells (purity > 97%) (Figure S2 in Supplementary Material) were either used directly as resting NK cells (rNKs) or furthered cultured in mouse medium (MM) containing 10% fetal bovine serum (FBS from Hyclone), 1% PSG (invitrogen), 1.6 mmol/l 2-ME, and IL-2 (1,000 U/ml) IL-2 concentration IL-2 (1,000 U/ml) for 4 days to obtain activated NK cells (aNKs). Sema-3E, Plexin D1, and NRP1 primers were designed by Integrated DNA Technologies Inc. Analysis total RNA was extracted from NK or DCs by using TRIzol (Invitrogen, Life Technologies, Cat #: 15596026, CA, USA) according to manufacturer’s protocol. RNA concentration was measured using BioPhotometer (Eppendorf AG, Hamburg, Germany). To synthesize cDNA, reverse transcription was perfumed with 2 µg of total RNA using a High-Capacity cDNA Reverse Transcription Kit (Applied Biosystems, USA, Cat #: 4368814) in a total volume of 20 µl according to the manufacturer’s protocol. cDNA of each sample and sequence specific Sema-3E Forward (5′–3′) (AAAGCATCCCCAACAAACTG) Reverse (5′–3′) (CTGGCTCGAGACCCTTACTG), Plexin D1 Forward (5′–3′) (TGGATGTCGCAGCTTACTTG) Reverse (5′–3′) (CCCCAACCCACAGTTCTCTA), NRP1 Forward (5′–3′) (TATTCCCAGAACTCTGCCC) Reverse (5′–3′) (TGTCATCCACAGCAATCCCA), and GAPDH Forward (5′–3′) (AACTTTGGCATTGTGGAAGG) Reverse (5′–3′) (ACACATTGGGGGTAGGAACA) primers (10 µM) were added to Master Mix (Applied Biosystems, USA, Cat #: 4472908). PCR was performed in small tubes using Master cycler gradient for 25 cycles. Real-time PCR was performed in 96-well optical plates with an initial 1 cycle denaturation step for 10 min at 95°C, 40 cycles of PCR (95°C for 15 s, 60°C for 35 s, and 72°C for 35 s), 1 cycle of melting and 1 cooling cycle (Applied Biosystems 7500 Real-Time PCR system). Average data collection and detection of fluorescent products were performed at the end of the 72°C extension period. Performing melting curve analysis and examining the quality of amplification curves assessed products specificity. Normalizing to the amplification of GAPDH calculated the amplification of target genes. Then the normalized values were expressed as fold increase/decrease of relative quantitative over the values calculated with other groups.

### Trans-Well Migration

Primary NK cells were isolated from splenocytes using the EasySep mouse NK negative selection kit (StemCell Technologies, Vancouver, BC, USA), according to the manufacturer’s protocol (Figure S3 in Supplementary Material). Activated isolated NK cells were cultured at 37 C and 5% CO_2_ in MM containing 10% FBS (from Hyclone), 1% PSG (Invitrogen), 1.6 mmol/l 2-ME, and IL-2 (1,000 U/ml). The purity of NK cells was checked before each experiment (Figure S3 in Supplementary Material). NK cell chemotaxis against MM, conditioned medium from iDC, LPS- or PIC- and mature DC were performed in the trans-well system. IL-2 aNKs (0.2 × 10^6^ in 100 µl) were loaded onto the upper chamber (5 µm pore Trans-well insert). The lower chamber contained either MM or conditioned media (600 µl). The cells were incubated at 37 C for 90 min. In some experiments, recombinant Sema-3E (at 6, 12, 25, 50, 100, or 200 ng/ml) was added to either the MM or conditioned medium. After 90 min, migrated NK cells were collected in the lower chamber, transferred to a polypropylene tube, centrifuged at 300 *g* for 10 min and counted under microscope absolute number of the migrated cells were calculated (32).

### Statistical Analysis

Data were analyzed statistically using GraphPad Prism (GraphPad Software, Inc.). Results were shown as the mean ± SEM. Two-tailed Student’s *t*-test was used in the statistical analyses of two data groups. One-way ANOVA was used in the statistical analyses of multiple data groups. A *p*-value of <0.05 was considered statistically significant. *p-value ≤ 0.05, **p-value ≤ 0.01, ***p-value ≤ 0.001, ****p-value ≤ 0.0001, NS (non significant) *p* value > 0.05.

## Results

### Expression Analyses of Sema-3E in DCs and NK Cells

**Figure 1:**
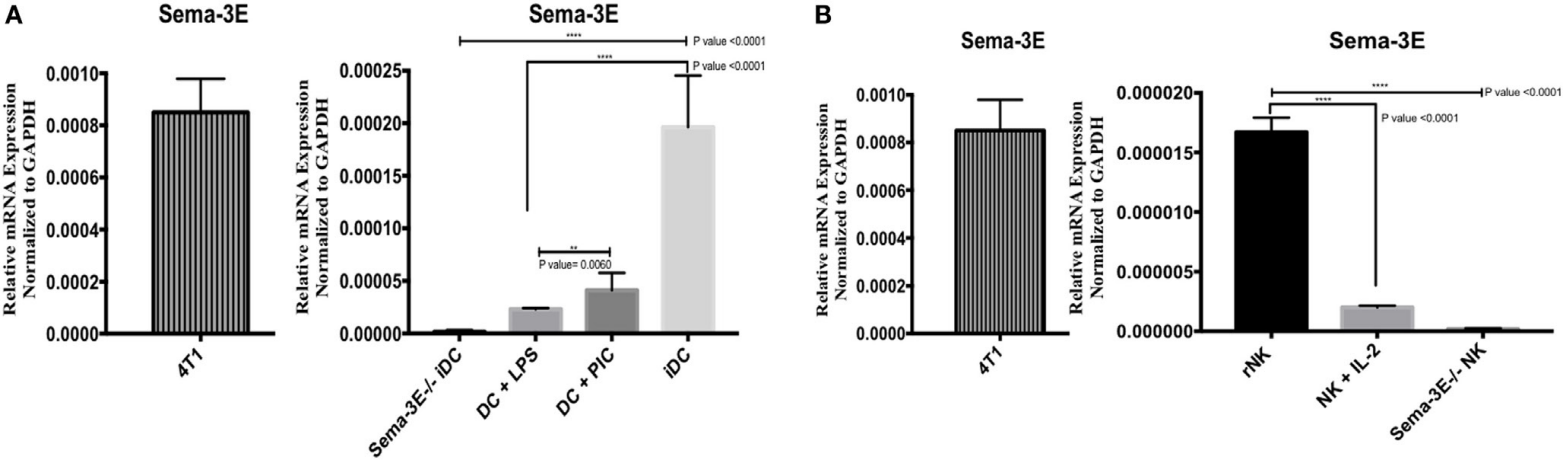
Expression of *Sema-3e* transcripts in dendritic cell (DC) and natural killer (NK) cells. Immature DCs (iDCs) were generated from BALB/c mice bone marrow by culturing the cells with GM-SCF for 7 days. DC were stimulated either by lipopolysaccharide (LPS) (1 µg/ml) or Poly:IC (PIC, 20 µg/ml) for 12 h. NK cells were sorted from the spleen of BALB/c mice, either used directly as resting NK cells (rNK) or cultured in the presence of IL-2 (1,000 U/ml) for 4 days (aNK). *Sema-3e^−/−^* iDC and *Sema-3e*^−/−^ NK were used as negative controls. 4T1breast cancer cell line was used as a positive control. The levels of *Sema-3e* were quantified using RT qPCR **(A,B)**. **(A)** The expression of *Sema-3e* in unstimulated iDC or DC stimulated by either LPS or PIC. **(B)** The expression of *Sema-3e* in rNK and IL-2 activated NK cells. One-way ANOVA followed by multiple comparison test was applied to compare between different groups at significance level of 0.05, *n* = 3/group. Two-tailed Student’s *t*-test was used to compare between two different groups at significance level of 0.05. Each experiment was carried out at least three times.

In the examination of the putative role of Sema-3E in NK-DC crosstalk, we first established the expression of Sema-3E mRNA in DC (immature, mature) and NK cells (“resting” *ex vivo*, IL-2 activated) by PCR. Total RNA was isolated from these cells for cDNA preparations. Sema-3E mRNA expression was analyzed by Sema-3E-specific primers in PCR assays. GAPDH was used as an internal control in all PCR analyses. Murine 4T1breast cancer cells, which expressed high level of Sema-3E (33), were used as positive control. DC or NK from the Sema-3E^−/−^ mice were used as negative controls. We demonstrated that the Sema-3E primers and the PCR conditions were Sema-3E-specific, as we detected no Sema-3E mRNA expression in the Sema-3E^−/−^ iDC cells (KO), whereas high level of Sema-3E mRNA expression was detected in the 4T1 cells (Figure 1A). Using this specific PCR condition for Sema-3E, we observed that high level of Sema-3E mRNA expression in immature or Poly I:C-activated mature Sema-3E^+/+^ DC (Figure 1A). Of interest, stimulation of the immature Sema-3E^+/+^ DC with LPS-induced downregulation of Sema-3E mRNA expression (Figure 1A). We examined also Sema-3E mRNA expression in the unstimulated *ex vivo* sorted “rNKs” and IL-2 aNKs. We detected low/no level of Sema-3E mRNA transcripts in both rNK and IL-2 aNK preparations (Figure 1B).

**Figure 2:**
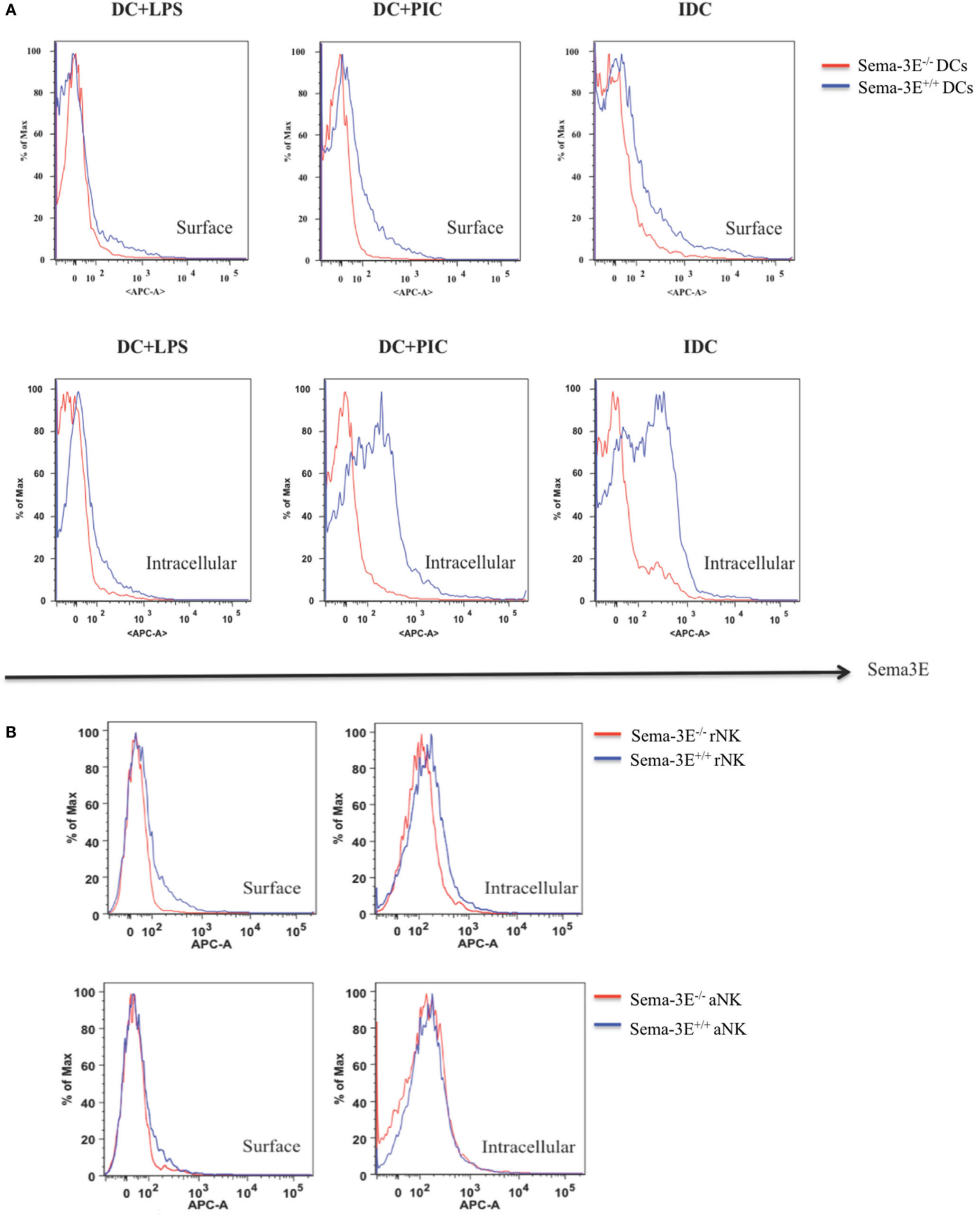

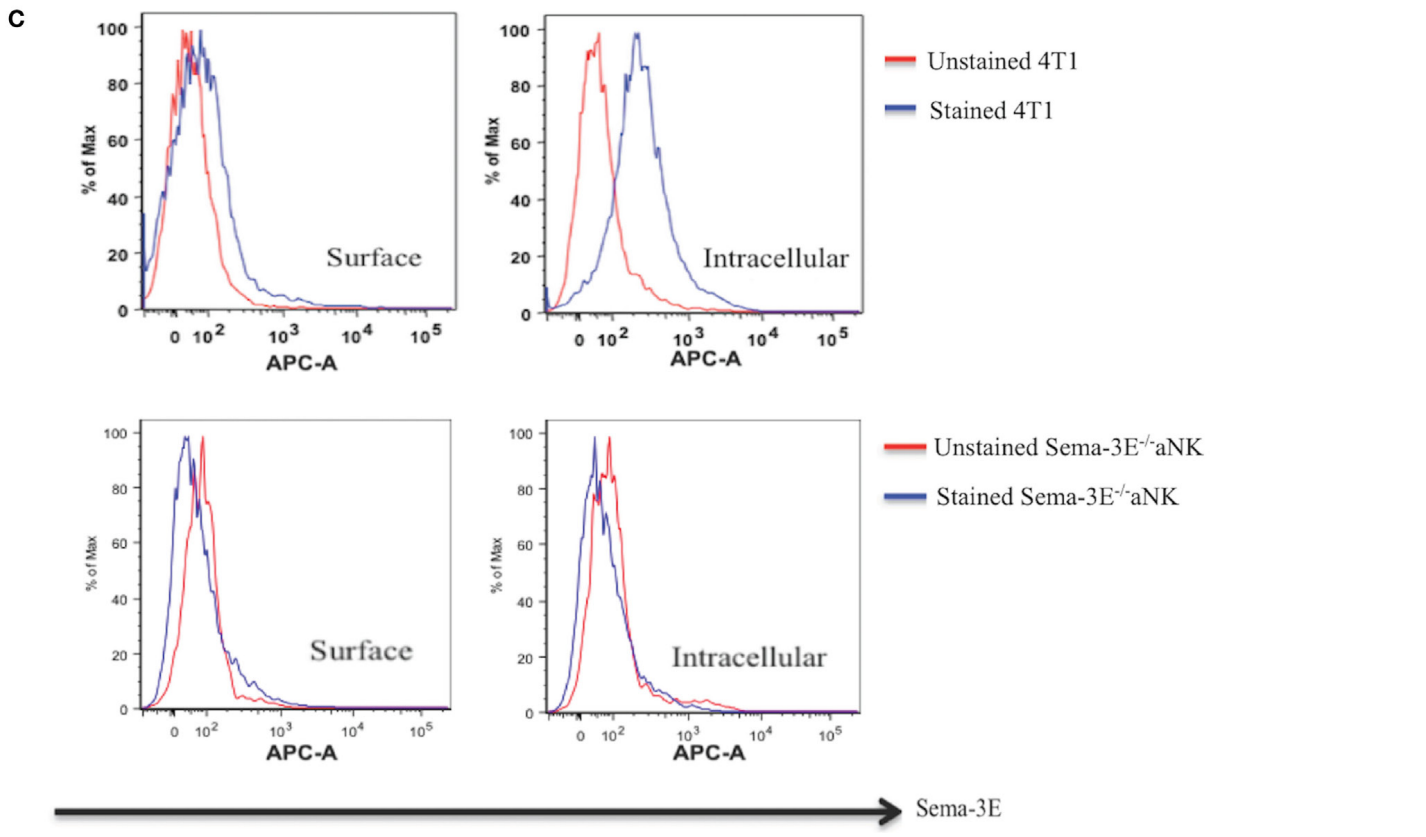
Intracellular and surface expression of semaphorin-3E (Sema-3E) proteins in dendritic cell (DC) and natural killer (NK) cells. Intracellular and/or surface expression of Sema-3E proteins on DC **(A)** or NK cells **(B)** were examined by APC anti-Sema-3E mAb in flow cytometry. Cell preparations of immature DC, DC stimulated by lipopolysaccharide (LPS) (DC + LPS) or DC stimulated by PIC (DC + PIC) either from Sema-3E^−/−^ (red line) or Sema-3E^+/+^ (blue line) mice were used. Sema-3E^−/−^ or Sema-3E^+/+^ resting NK (rNK) and IL-2 activated NK cells (aNKs) were used. NK or DC cells from Sema-3E^−/−^ mice were used as negative controls. 4T1 breast cancer cell line was used as a positive control **(C)**. Unstained (red line) and stained Sema-3E^−/−^ aNK cells (blue line) were used for Sema-3E mAb specificity **(C)** (*n* = 3 independent experiments).

To corroborate with our mRNA analyses, we used Sema-3E-specific antibodies to detect the Sema-3E protein expression in flow cytometry. No Sema-3E proteins were detected either by surface staining (for surface Sema-3E) or intracellular staining (for both surface and intracellular Sema-3E) when cells from Sema-3E^−/−^ mice were used. High levels of Sema-3E protein expression were detected in the 4T1 (positive control) breast cancer cells, iDC, and poly I:C-activated mature DC (Figures 2A,C). In agreement with the mRNA expression data, Sema-3E protein expression was downregulated in the LPS-activated mature DC (Figure 2A). We observed low/no level of Sema-3E protein expression in rNK and IL-2 aNKs (Figure 2B).

### Expression Analyses of Plexin D1 and Neurophilin Receptors in DCs and NK Cells

**Figure 3:**
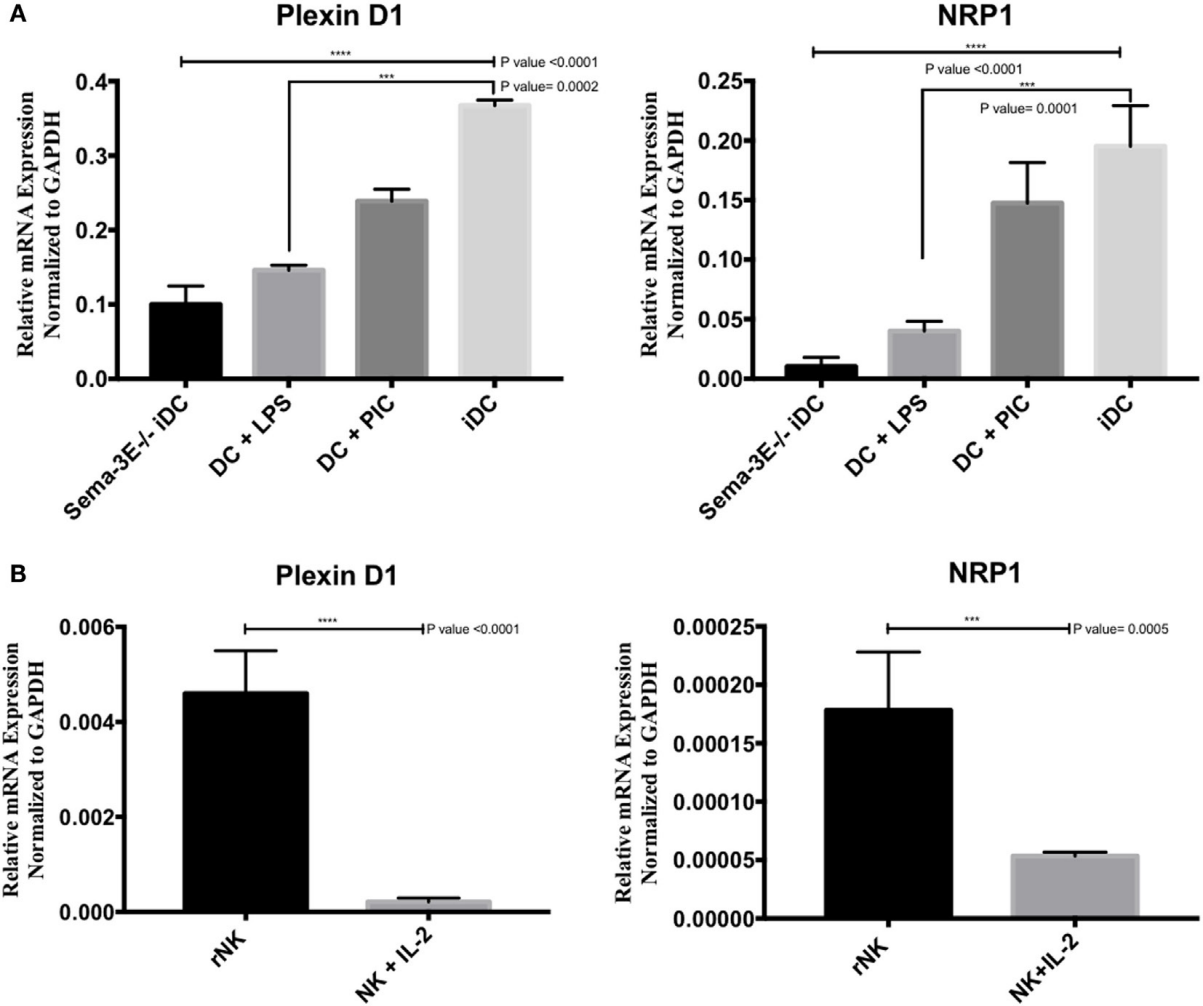
Expression of *Plexin D1* and *NRP1* receptors in dendritic cell (DC) and natural killer (NK) cells. Immature DCs (iDCs) were generated from BALB/c mice bone marrow by culturing the cells with GM-SCF for 7 days. DC were stimulated either by lipopolysaccharide (LPS) (1 µg/ml) or Poly:IC (PIC, 20 µg/ml) for 12 h. NK cells were sorted from the spleen of BALB/c mice, either used directly as resting NK cells (rNK) or cultured in the presence of IL-2 (1,000 U/ml) for 4 days (activated NK cell). The levels of *Plexin D1* and *NRP1* were quantified using RT qPCR **(A,B)**. **(A)** The expression of *Plexin D1* and *NRP1* in unstimulated iDC or DC stimulated by either LPS or PIC. **(B)** The expression of *Plexin D1* and *NRP1* in rNK cells and IL-2 activated NK cells. RNase-free water used as negative specificity control. One-way ANOVA followed by multiple comparison test was applied to compare between different groups at significance level of 0.05, *n* = 3/group **(A)**. Each experiment was carried out at least three times. Two-tailed Student’s *t*-test was used to compare between different groups at significance level of 0.05 (*n* = 3 independent experiments) **(B)**.

To investigate the expression of Plexin D1 (Sema-3E receptor) and NRP1 (Sema-3E co-receptor) receptors in DC and NK cells, we isolated mRNA from iDC, mature DC (LPS-activated or Poly I:C activated), rNK-cell, and IL-2 aNK cultures. cDNA preparations were used in PCR using Plexin D1- and NRP1-specific primers. The Plexin D1 and NRP1 primers and PCR conditions were specific, as no Plexin D1/NRP1 mRNA expressions were detected in the negative control (RNA’s Free Water). In contrast, high level of Plexin D1 and NRP1 mRNA expressions were detected in immature or Poly I:C- stimulated Sema-3E^+/+^ DC cells (Figure 3A). Stimulation of the immature Sema-3E^+/+^ DC with LPS resulted in downregulation of PlexinD1 and NRP1 mRNA expressions (Figure 3A). Of interest, we also observed that Plexin D1 and NRP1 mRNA expressions were downregulated significantly in the Sema-3E^−/−^ iDC when compared with the Sema-3E^+/+^ iDC (Figure 3A). For NK cells, we observed expression of Plexin D1 and NRP1 mRNA in the highly purified rNK cells (Figure 3B). However, their expression levels were much lower than that of iDC and activated DC (Figures 3A,B). Upon IL-2 activation, Plexin D1 and NRP1 mRNA expressions were downregulated significantly in the IL-2 aNKs (Figure 3B).

**Figure 4:**
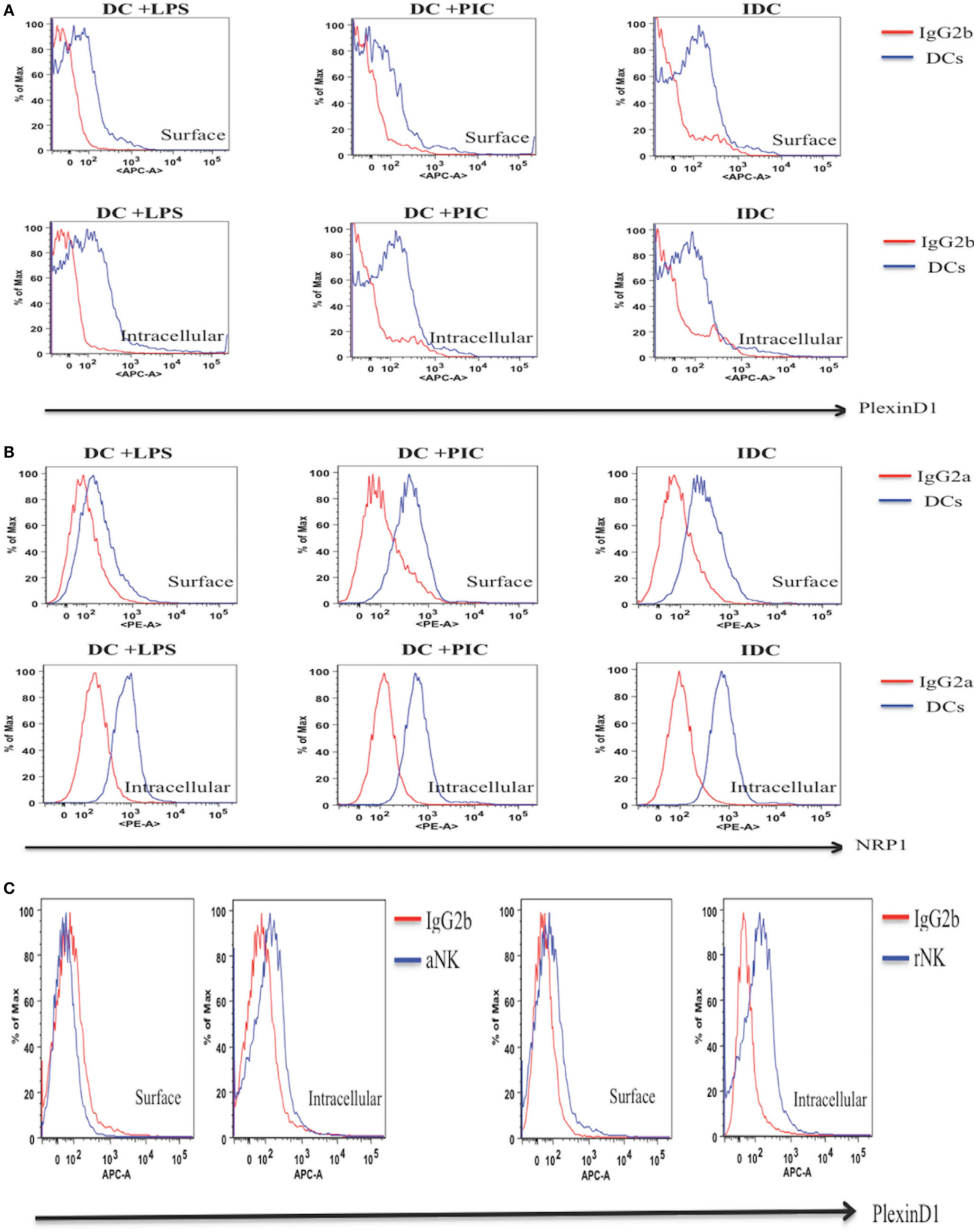

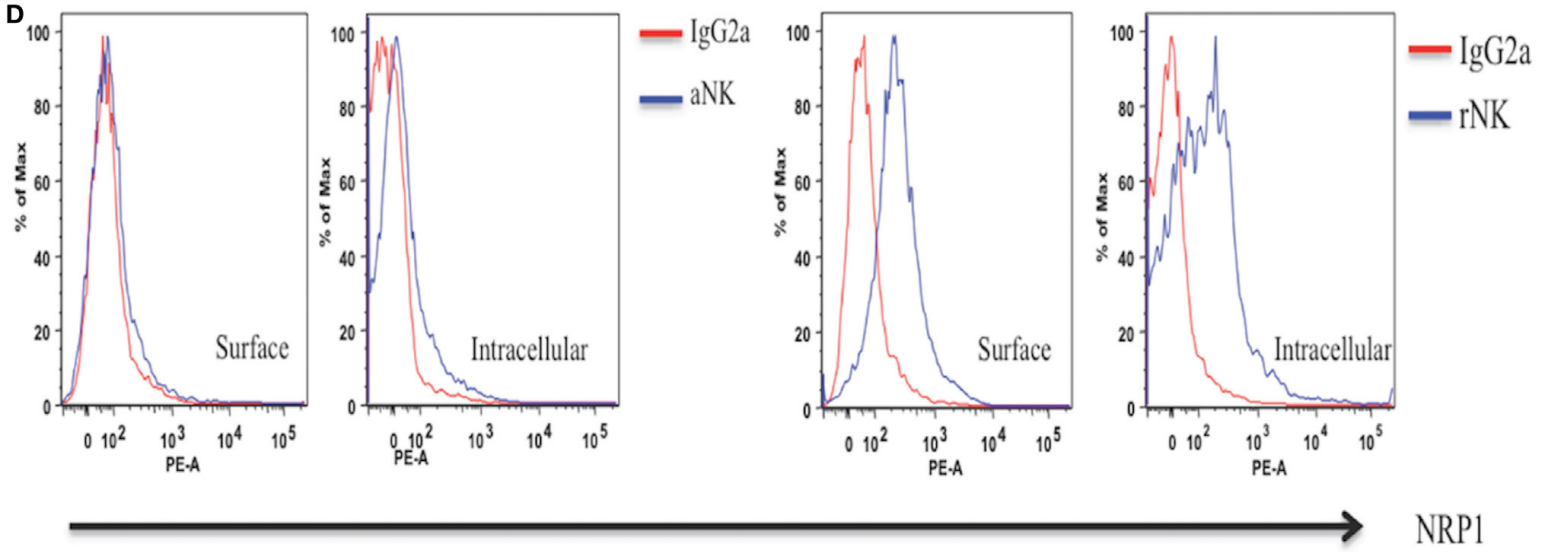
Intracellular and surface expression of Plexin D1 and NRP1 receptor proteins in dendritic cells (DC) and natural killer (NK) cells. Intracellular and/or surface expression of Plexin D1 and NRP1 receptor proteins on DC **(A,B)** or NK cells **(C,D)** were examined by respective APC anti-Plexin D1 and PE anti-NRP1 mAbs in flow cytometry. Semaphorin-3E (Sema-3E)^+/+^ cells preparations of immature DC, DC stimulated by lipopolysaccharide (LPS) (DC + LPS) or DC stimulated by PIC (DC + PIC) were used (blue line). Sema-3E^+/+^ resting NK (rNK) and IL-2 activated NK cells (aNK) were used (blue line). Isotype control mAbs (IgG2b for Plexin D1, IgG2a for NRP1) were used as negative controls (red line). **(A)** Expression of Plexin D1 on DC. **(B)** Expression of NRP1 on DC. **(C)** Expression of Plexin D1 on NK. **(D)** Expression of NRP1 on NK (*n* = 3 independent experiments).

Next, we used anti-Plexin D1 or -NRP1 mAbs in flow cytometry to detect protein expression of these receptors on iDC, mature DC, rNK, and IL-2 aNKs. Isotype controls for anti-Plexin D1 (IgG2b) and anti-NRP1 (IgG2a) were used as negative controls for Plexin D1 and NRP1 staining, respectively. In agreement with the mRNA expression data, both Plexin D1 and NRP1 receptors were detected in DC (iDC, LPS-activated, and PIC-activated) and NK cells (resting and activated) in intracellular flow cytometry (Figure 4). Expression of these receptor proteins on DC seems to be higher than that of NK cells (Figures 4A and 4D). We observed downregulation of the protein expressions of the Plexin D1 and/or NRP1 receptors in the LPS-activated DC and IL-2 aNKs (Figure 4).

### Recombinant Sema-3E Alone Did Not Affect NK-Cell Migration in Plain Complete Mouse Culture Medium in a Trans-Well Assay

**Figure 5:**
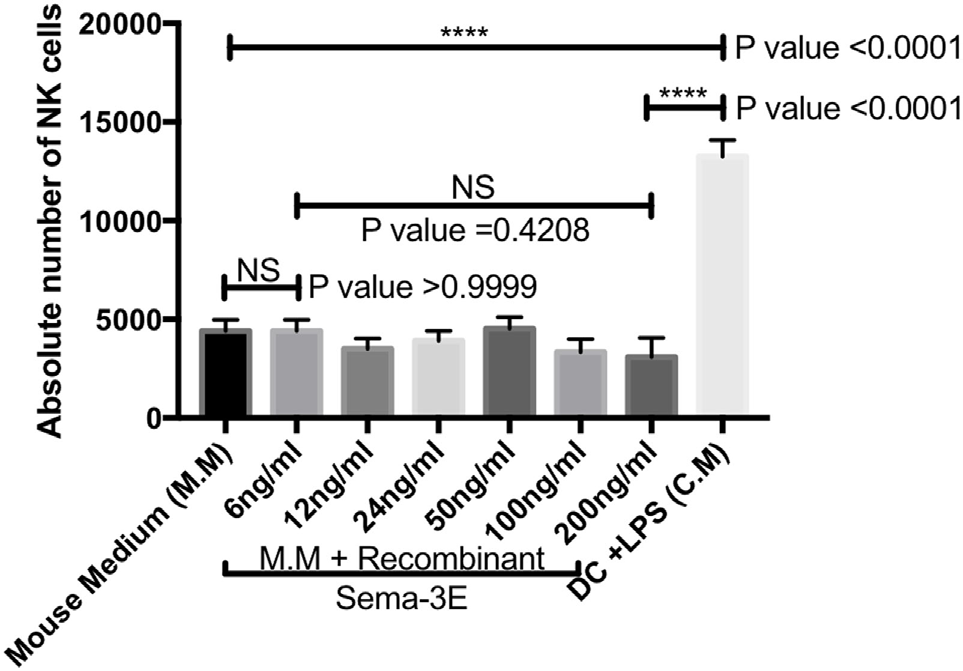
Recombinant semaphorin-3E (Sema-3E) alone did not affect migrations of activated natural killer (NK)-cell migration in plain mouse culture medium: NK cells were purified and activated with IL-2 for 4 days. Sema-3E was added to plain mouse medium (MM) using at different concentrations (6–200 ng/ml). Plain MM was used as negative control. Conditioned medium from lipopolysaccharide (LPS)-stimulated dendritic cell (DC) was used as positive control. Activated NK-cell migration was assayed in the trans-well chamber. One-way ANOVA was used for comparing data from more than two groups. Two-tailed Student’s *t*-test was used to compare between different groups at significance level of 0.05. The statistical analysis was obtained from *n*-value = 3.

We have previously reported that conditioned medium from iDC and mature DC regulated chemotaxis of aNKs (34). aNKs expressed receptors for Sema-3E (Figures 3B and 4C,D). Sema-3E was expressed in iDC (Figures 1A and 2A). We therefore examined whether Sema-3E alone could regulate IL-2-aNK migration in plain MM in a trans-well assay *in vitro*. IL-2 aNKs were loaded on the upper chamber in the trans-well, whereas plain complete mouse culture medium was placed in the lower chamber. Different concentrations of recombinant Sema-3E (6–200 ng/ml range) were added to the complete MM to examine whether Sema-3E promoted NK-cell migrations. NK-cells migrated to the lower chamber were counted at the end of the assay. We used plain complete MM and conditioned medium from the LPS-activated DC as negative and positive controls in the assay. As expected, we observed strong chemotactic migrations of NK cells in the presence of the conditioned medium from LPS-activated DC, when compared with that of the plain complete MM control (*P* < 0.0001). The presence of various concentration of recombinant Sema-3E, however, did not seem to affect the low level of basal NK-cell migrations in plain mouse culture medium (Figure 5).

### Sema-3E Produced by iDC Suppressed NK-Cell Migration *In Vitro*

**Figure 6:**
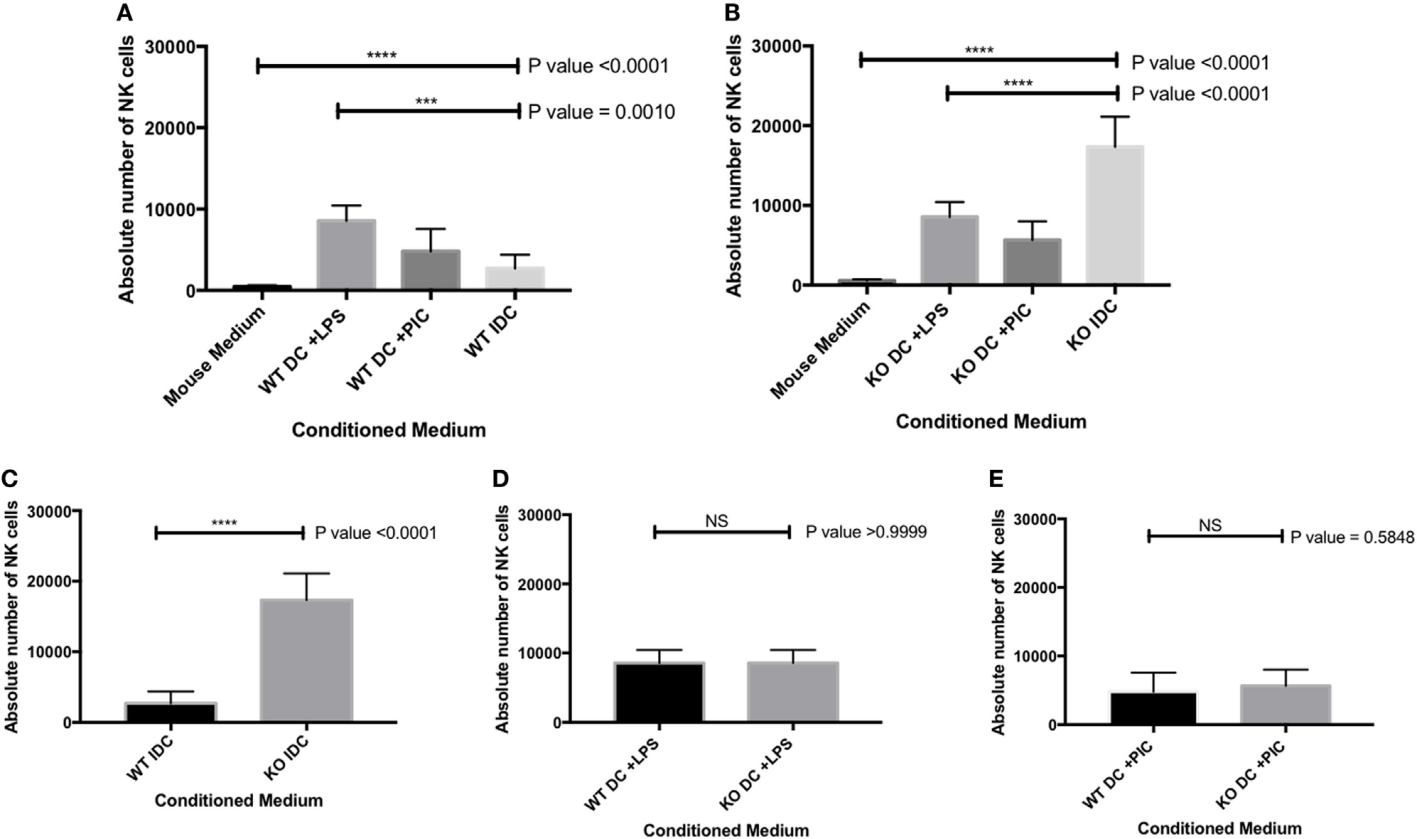
Semaphorin-3E (Sema-3E) produced by immature dendritic cell (iDC) modulated activated natural killer (NK)-cell migration: migrations of the IL-2 activated NK cells (aNKs) toward different DC conditioned media were examined in the trans-well migration assay. Conditioned media of the Sema-3E^+/+^ and Sema-3E^−/−^ iIDC, mature lipopolysaccharide (LPS)-stimulated DC (DC + LPS), mature PIC-stimulated DC (DC + PIC) were collected on day 8 of the DC cultures. **(A)** NK-cell migrations toward the conditioned media of the Sema-3E^+/+^ DC. **(B)** NK-cell migrations toward the conditioned media of the Sema-3E^−/−^ DC. **(C–E)** Direct comparisons of the aNK migrations in various conditioned media of the Sema-3E^+/+^ (WT) DC and Sema-3E^−/−^ (KO) DC: **(C)** conditioned media of the iDC. **(D)** Conditioned media from the LPS-stimulated DC. **(E)** Conditioned media from the Poly I:C-stimulated DC. One-way ANOVA was used for comparing data from three or more groups **(A,B)** and two-tailed Student’s *t*-test was used to compare between two different groups at significance level of 0.05, *n* = 3/group **(C,D,E)**. Each experiment was carried out at least three times.

We showed that iDC and mature DC (stimulated with either LPS or Poly I:C) expressed various Sema-3E at mRNA and protein levels (Figures 1A and 2A). We therefore used the trans-well assay to investigate whether DC-derived Sema-3E could regulate NK-cell migration in the context of NK-DC crosstalk. IL-2 aNKs were loaded on the upper chamber in the trans-well, whereas complete MM or conditioned medium from the iDC and mature (LPS, Poly I:C-activated DC) of the Sema-3E^+/+^ and Sema-3E^−/−^ DC were placed in the lower chamber. NK-cells migrated to the lower chamber were counted at the end of the assay. LPS or Poly I:C stimulated mature Sema-3E^+/+^ DC promotes aNK migration (*P* < 0.0001) (Figure 6A). Conditioned medium from immature Sema-3E^+/+^ DC induced a weaker NK-cell migration when compared with that of the mature Sema-3E^+/+^ DC (Figure 6A) (*P* = 0.0010). Using this established protocol, we next used DC from Sema-3E^−/−^ mice to examine the impact of DC-derived Sema-3E on NK-cell migrations (Figure 6B). Strikingly, we observed that conditioned medium from the immature Sema-3E^−/−^ DC promoted a stronger aNK chemotactic response, when compared with that of the LPS- or Poly I:C-activated Sema-3E^−/−^ DC (Figure 6B) (*P* < 0.0001). A direct comparison of the NK-cell migratory responses toward conditioned media from the Sema-3E^+/+^ and Sema-3E^−/−^ iDC or activated DC showed the enhanced NK-cell migrations in the conditioned medium from the immature Sema-3E^−/−^ DC, when compared with that of the immature Sema-3E^+/+^ DC (Figure 6C) (*P* < 0.0001). No significant migratory differences were observed when the conditioned media from the LPS-, poly I:C-activated Sema-3E^+/+^ or Sema-3E^−/−^ DC were used (Figures 6D,E).

**Figure 7:**
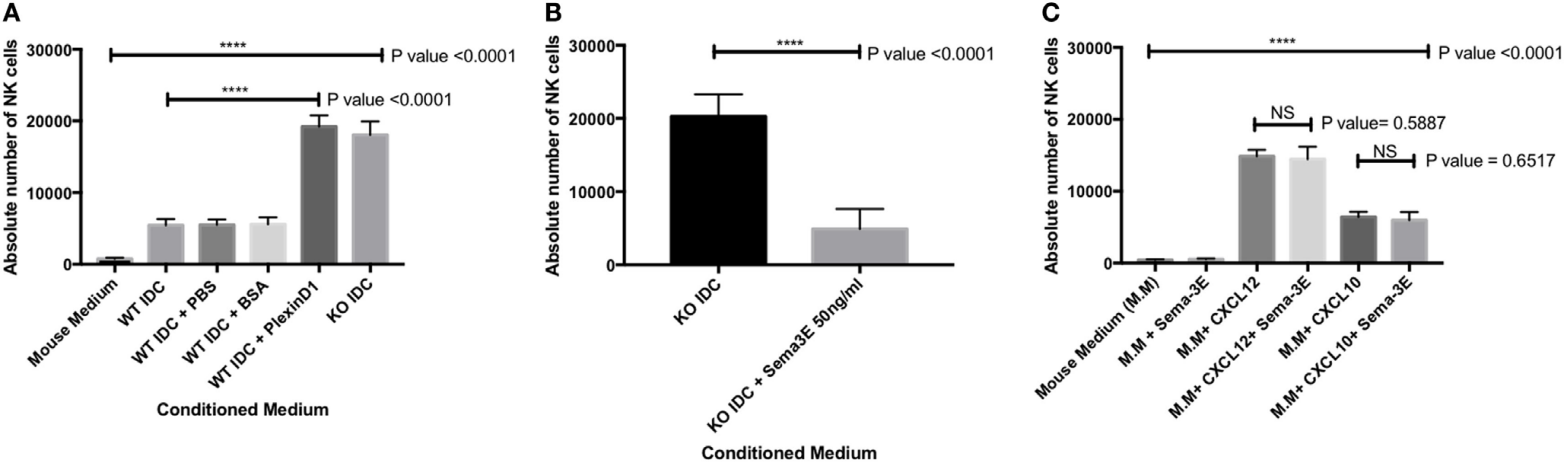
Blocking of semaphorin-3E (Sema-3E) produced by immature dendritic cell (iDC) enhanced activated natural killer (NK) cells migration: migrations of the IL-2 activated NK cells toward the conditioned medium of the Sema-3E^−/−^ (KO IDC) and Sema-3E^+/+^ (WT IDC) iDC were assayed. **(A)** Recombinant Plexin D1 (100 ng/ml) was added to the WT IDC conditioned medium at the time of running the migration assay. 1% PBS and 1% BSA (100 ng/ml) were used as negative controls. Conditioned medium of the Sema-3E^−/−^ (KO IDC) was used as positive control. **(B)** The introduction of recombinant Sema-3E back to the conditioned medium of the Sema-3E^−/−^ iDC abrogated the enhanced NK-cell migration. Recombinant Sema-3E (50 ng/ml) was added to the KO IDC conditioned medium at the time of running the migration assay. **(C)** Sema-3E has no regularity effect on NK-cell migration through CXCL12 or CXCL10 chemokines. CXCL12 chemokine (50 ng/ml) and CXCL10 chemokine (100 ng/ml) were added to mouse medium at the time of running the migration assay. Statistical significance was established by one-way ANOVA was obtained to establish the comparison between three or more groups **(A,C)**. Two-tailed Student’s *t*-test was used to compare between two indicated groups at significance level of 0.05 **(B,C)** (*n* = 3 independent experiments).

We confirmed that the enhanced NK-cell migration we observed in the conditioned medium from the immature Sema-3E^−/−^ DC was Sema-3E-specific. First, we used recombinant Plexin D1 receptor protein (35) as decoy receptors to remove soluble Sema-3E in the conditioned medium from the immature Sema-3E^+/+^ DC. Recombinant Plexin D1 receptor proteins or control proteins (BSA) were added into the conditioned medium from the immature Sema-3E^+/+^ DC conditioned medium at the time of the trans-well assay. We observed no differences in the migratory responses of the IL-2 aNKs toward conditioned media from the immature Sema-3E^+/+^ DC, whether PBS or BSA was used. However, “blocking” of Sema-3E in the conditioned medium of the immature Sema-3E^+/+^ DC enhanced NK-cells migration we observed (*P* < 0.0001) (Figure 7A). Second, we added recombinant murine Sema-3E protein to the conditioned medium from the immature Sema-3E^−/−^ DC at the time of the trans-well assay. We observed that re-introduction of recombinant Sema-3E to the conditioned medium of the immature Sema-3E^−/−^ DC abrogated the enhanced NK-cells migration observed (*P* < 0.0001) (Figure 7B). Addition of recombinant Sema-3E to the conditioned medium of the LPS-mature Sema-3E DC had no effect on NK-cell migration (Figure S4 in Supplementary Material).

CXCL12 and CXCL10 chemokines have been reported to play an important role in regulating immune cells recruitment (36, 37). We therefore used the trans-well assay to investigate whether Sema-3E could regulate NK-cell migration through CXCL12 or CXCL10 chemokines. Recombinant CXCL12 (50 ng/ml) or CXCL10 (100 ng/ml) was added to MM plus or minus recombinant murine Sema-3E protein (50 ng/ml). We observed, however, no effects of Sema-3E on NK migration induced by these single chemokines (Figure 7C; Figures S5 and S6 in Supplementary Material).

## Discussion

In this study, we first examined RNA and protein expressions of Sema-3E, Plexin D1, and NRP1 in primary NK and DC. We reported that NK cells expressed receptors for Sema-3E, and that Sema-3E was expressed in iDC, thus establishing the relevance of studying Sema-3E in NK-DC crosstalk. Next, we investigated the effect of Sema-3E in NK-cell migration. We observed that recombinant Sema-3E alone did not affect the low level of “basal” NK-cell migration in plain mouse media. Conditioned medium of the mature Sema-3E^+/+^ DC promoted a stronger chemotaxis of the IL-2 aNKs than the immature Sema-3E^+/+^ DC conditioned medium in trans-well assay. Interestingly, enhanced NK-cell migration was observed in the conditioned medium of the immature Sema-3E^−/−^ DC when compared with the conditioned medium of the immature Sema-3E^+/+^ DC. Addition of the exogenous recombinant Sema-3E protein to the conditioned medium of the immature Sema-3E^−/−^ DC abrogated the enhanced NK-cell migration observed, thus supporting a suppressive effect of Sema-3E in iDC in regulating NK-cell migration.

Natural killer and DC are capable of secreting a wide range of cytokines and chemokines that regulates diverse recruitment and functions of other immune cells (38, 39). Of particular interest to us, interactions of NK and DC are also bi-directional. By means of target cell recognitions, different cytokines stimulation (such as IL-2, IL-12, IL-15, and IL-18) (9–12), and DC interactions in the microenvironments, NK-cell activation, and function are regulated (13, 14). For example, DC-derived cytokines such as IL-12 and IL-18 are critical, respectively, in the generation of IFN-γ producing NK cells and the acquisition of IL-12-receptor on NK cells (40). Mutually, NK cells can induce DC maturation by upregulating expression of molecules (such as MHC and CD86) and DC activation *via* Triggering Receptor Expressed on Myeloid Cells 2 and NKp30 signaling to enhance their ability to secrete IL-18, IL-12 (18, 41). The importance of NK-DC crosstalk in coordinating anti-tumor and anti-microbial responses *in vivo* has been well established (42, 43); however, the molecular regulation of NK-DC crosstalk in steady state has not been fully elucidated.

Semaphorin-3E is an axon-guidance secreted protein in the neuronal system that has been reported to be essential in regulating neural cell migration and proliferation (21). Recent work, however, has established its multi-functional roles in other biological processes that range from cancers, autoimmunity, and allergic diseases (44). In this report, we focused on the ability of Sema-3E in regulating NK-cell migration, particularly in NK–DC interactions. We first examined any direct effect of Sema-3E alone on aNK migration. It was noted that recombinant Sema-3E alone had no effect on aNK migration in trans-well assay (Figure 5). It thus suggested that Sema-3E might exert its regulatory effect on NK-cell migration, if any, in the presence of other chemotactic factors. iDC and LPS-activated mature DC released soluble factors, which induced different level of chemotactic movement of IL-2-aNKs *in vitro* (34). We therefore used DC derived from the Sema-3E-proficient (WT, Sema-3E^+/+^) and Sema-3E-deficient (Sema-3E^−/−^) mice to examine the impact of Sema-3E derived from iDC, LPS-activated or Poly I:C-activated DC on the regulation of aNK migration in trans-well assays. Deficiency of Sema-3E in the conditioned medium of the iDC (but not LPS- or Poly I:C-activated DC) derived from the Sema-3E^−/−^ mice, resulted in a significant increase in aNK migration (Figure 6B). Recent studies in cultured cells have shown that Plexin D1 itself displayed R-Ras GAP activity to inhibit migration, and these actions specifically required the small GTPase Rnd2 (27). In addition, Rnd2 was found to bind to Plexin D1 in cortical neurons, and Sema-3E-Plexin D1-induced inhibition of axon outgrowth of cortical neurons required Rnd2 and downregulation of R-Ras activity (45). We showed that NK cells expressed Plexin D1 and NRP receptors (Figures 3 and 4). The relatively low expressions of the Plexin D1 and NRP1 receptor proteins in NK cells could be a result of a lower cross-reactivity of the commercially available antibodies and/or the need of a tight regulation of these receptors on rNK or aNKs. Nevertheless, we speculated that Sema-3E derived from iDC exerted its suppressive effect on aNK migration *via* Plexin D1/NRP signaling. The latter might intersect other chemokine receptor(s) and Ras signaling pathways that were induced upon engagements of chemokines produced by iDC. The use of Plexin D1-deficient NK cells in future will formally establish whether the effect of Sema-3E on aNK migration is induced by the cognate Sema-3E-Plexin D1 signaling pathway.

The apparently different effects of the Sema-3E derived from different maturation states of DC in the regulation of aNK migration may lie onto two possible factors: first, the tight regulation of Sema-3E in different maturation states of DC. High level of Sema-3E expression was observed in iDC. Upon further LPS stimulation, Sema-3E expression was downregulated in these LPS-activated mature DC. Second, the chemotactic factors produced by DC at different maturation states induced by different TLR ligands (such as LPS and Poly I:C) may collectively present a differential environment that has different sensitivity toward the suppressive effect of Sema-3E exerted on the aNKs. Toward this end, we observed that recombinant Sema-3E had no suppressive effect on the migratory responses of aNKs toward conditioned medium from LPS-mature DC (Figure S4 in Supplementary Material), as well as mouse media containing single chemokine (such as CXCL10, CXCL12, Figure 7C; CCL19, CCL21, Figures S5 and S6 in Supplementary Material). Future studies are required to delineate the unique chemokine(s) produced by iDC that is sensitive to Sema-3E suppression in NK-cell migrations.

Using Sema-3E-deficient and Plexin-D1-deficient mouse models, respectively, we and others have previously revealed a critical immune regulatory role of Sema-3E/Plexin D1 in DC phenotypes and experimental allergic asthma (28, 29). We used Sema-3E-deficient mice in experimental models of asthma to examine the role of Sema-3E in the development and maintenance of allergic asthma. We observed Th2/Th17 lung inflammation, increased airway hyper-responsiveness, and increased lung granulocytosis in the Sema-3E-deficient mouse model (28, 29). The role of Sema-3E in regulating allergic asthma seems to associate with the recruitment of pulmonary DC subset and neutrophils (28, 29). It is possible that the absence of Sema-3E in steady state DC promote migrations and/or interactions of NK cells, which in turn impacts on the maturation states of these Sema-3E-deficient DC and subsequent immune responses upon allergen challenges. Holl et al. reported the predominant expression of the Plexin D1 receptor on myeloid DC and bone marrow-derived DC, and high level of Sema-3E expression in splenic DC (29). No observable differences in LPS-induced maturation and migratory responses toward CXCL12/19 gradients were observed when the Plexin D1-deficient and wild-type DC were compared. Interestingly, the Plexin D1-deficient DC, when compared with the wild-type DC, produced higher level of IL-12/IL-23 p40 (but not IL-6) when these sorted splenic DC were cultured *in vitro* at the steady state for 12 h (29). As IL-12 is critical to NK-cell activation, it will be interesting to examine how Plexin D1/Sema-3E signaling in DC regulates NK-cell activation/functions indirectly *via* IL-12. Our current Sema-3E expression data in iDC and mature DC (Figure 1), as well as the significant downregulation of the mRNA expression of Plexin D1 and NRP1 receptors in the immature Sema-3E^−/−^ DC (Figure 3), suggested also a possible “autocrine” feedback regulation of DC maturation/functions.

In conclusion, our current work demonstrated, for the first time, a novel role of iDC-derived Sema-3E in the regulation of aNK migration. We demonstrated that mouse NK cells expressed Sema-3E receptors, and that these receptors were tightly regulation upon activation. It thus suggests other direct regulatory effects of Sema-3E on NK and/or aNK functions. Future work will establish how such regulations, in NK-DC crosstalk, may affect DC maturation and homeostasis in steady state and disease models *in vivo*. Understanding the role of Sema-3E in regulating NK-cells function(s) may provide novel therapeutic targets to manipulate specific NK-cell effector functions and/or migration in clinical settings.

## Ethics Statement

This study was carried out in accordance with the recommendations of the Canadian Council for Animal Care. The protocol was approved by the Research ethics boards of the University of Manitoba, Winnipeg, Canada (protocol # 13-018).

## Author Contributions

AbdulazizA, AG, and SK conceived and designed the experiments, interpreted the data, and wrote the manuscript. AbdulazizA performed the experiments and analyzed the data. MZ and AbdulazizA: flow sorting of NK cells. AbdulazizA, RR, and AbeerA: PCR data. All authors have read and approved the manuscripts.

## Conflict of Interest Statement

The authors declare that the research was conducted in the absence of any commercial or financial relationships that could be construed as a potential conflict of interest.
