# Supplementary material for: Semaphorin-3E Produced by Immature Dendritic Cells Regulates Activated Natural Killer Cells Migration
Source: Front Immunol. 2018 May 9;9:1005. doi: 10.3389/fimmu.2018.01005 (PMC5954025; doi:10.3389/fimmu.2018.01005)
Supplement: Supplementary file 1 [file data_sheet_1.DOCX]

**Supplementary Materials**

**Semaphorin-3E produced by immature dendritic cells regulates activated** **natural killer cells migration**

Running Title: Semaphorin-3E & innate immunity

Abdulaziz Alamri^1^, Rahmat Rahman^1^, Manli Zhang^1^, Abeer Alamri^2^, Abdelilah S. Gounni^1^, Sam K.P. Kung^1*^

1: Department of Immunology, University of Manitoba, Winnipeg, MB, Canada

2: Department of Oral Biology, University of Manitoba, Winnipeg, MB, Canada

*: Corresponding Author

Corresponding: Dr. **Sam K.P Kung.**

Email: [Sam.Kung@umanitoba.ca](mailto:Sam.Kung@umanitoba.ca)

**keywords:** Semaphorins, Innate Immunity, Cell trafficking, Cytokines Activation, TLR-ligands, Natural Killer cells, Dendritic cells

**S1**

**
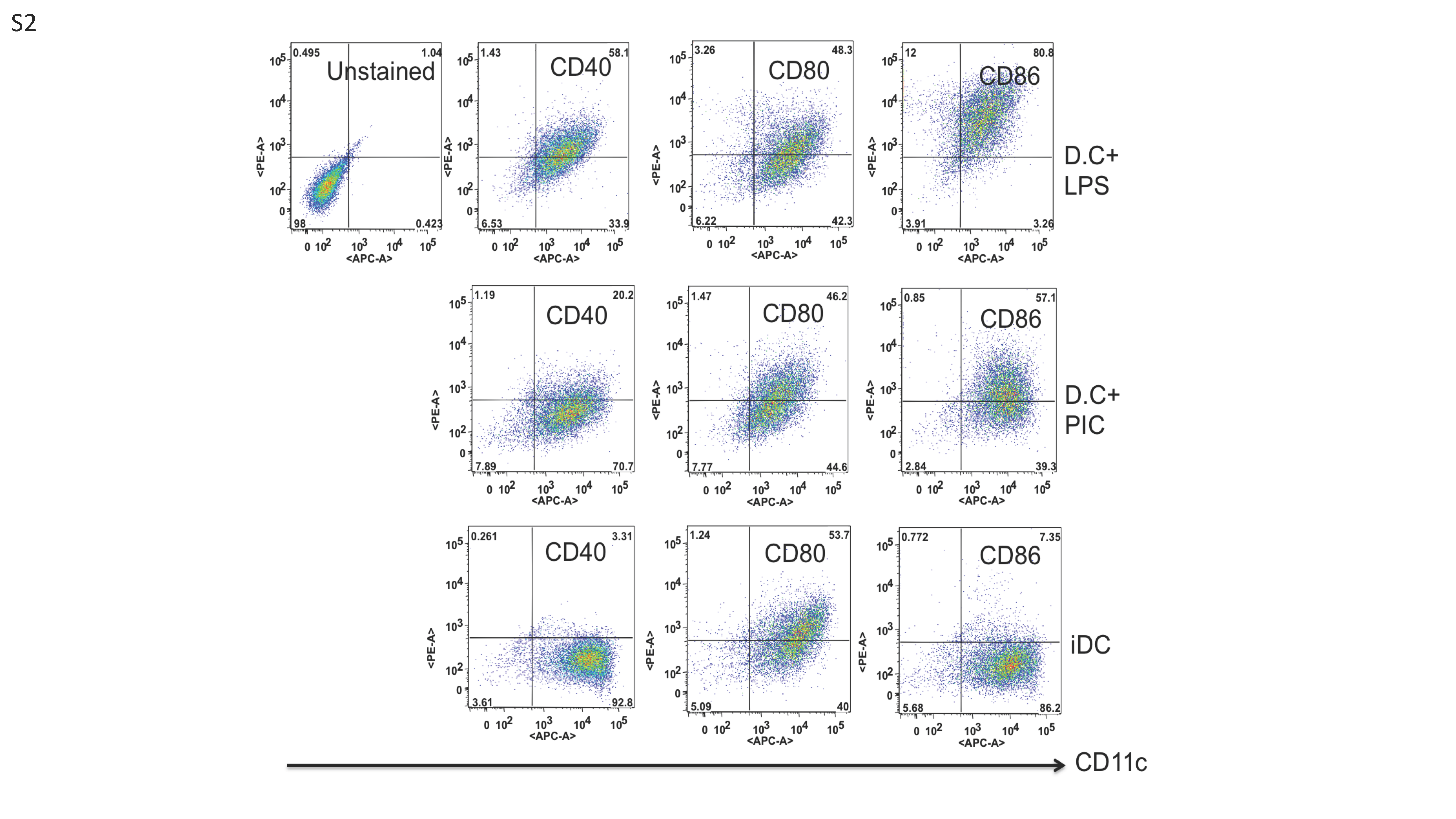
**

**Figure S1. Phenotypes of bone marrow derived dendritic cells preparations.** BMDCs were cultured in GM-CSF medium. On day-8, lipopolysaccharide (LPS (1μg/μl) and/or Polyinosinic:polycytidylic acid (Poly I:C) 20 μg/μl were introduced in the culture for 12-hours to acquire matured DC-phenotype. Surface staining was used to detect the expression of CD40, CD80 and CD86 surface markers, which represent matured-DC phenotype. In contrary to immature DCs (iDC), mature DCs with LPS up-regulated mature –DC marker.

**S2**

**
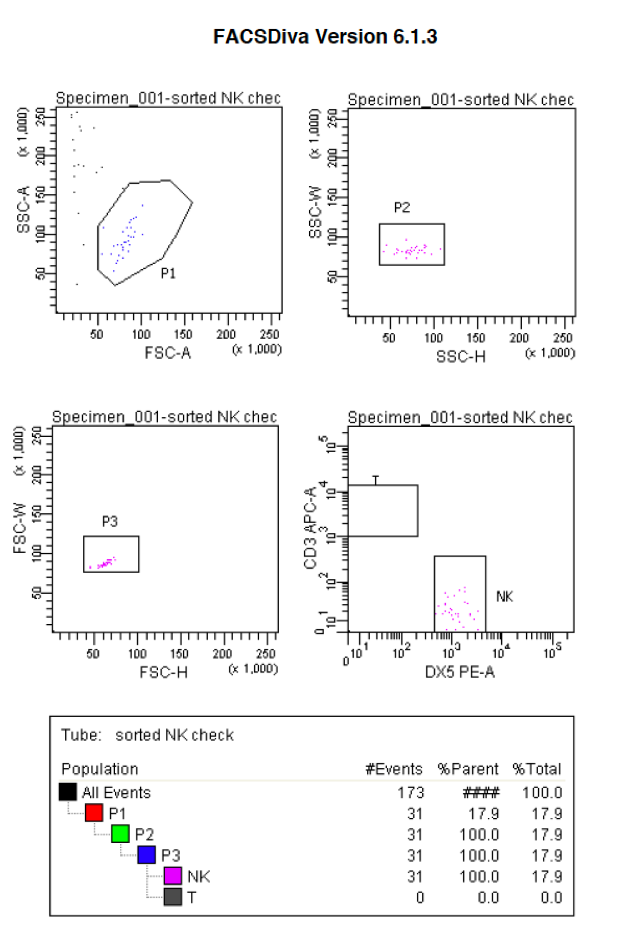
**

**Figure S2. Purity of the FACSorted NK cells used in the qPCR analyses.** NK cells were sorted from mouse spleen by FACSDiva using DX5^+^, CD3^-^ monoclonal antibodies as described in Materials and Methods. Sorted NK cells were either used as resting NK cells immediately or further activated in IL-2 (1000U/ml) for 4 days. We obtained 97-100% purity in these FACSorted NK cells.

**S3**

**
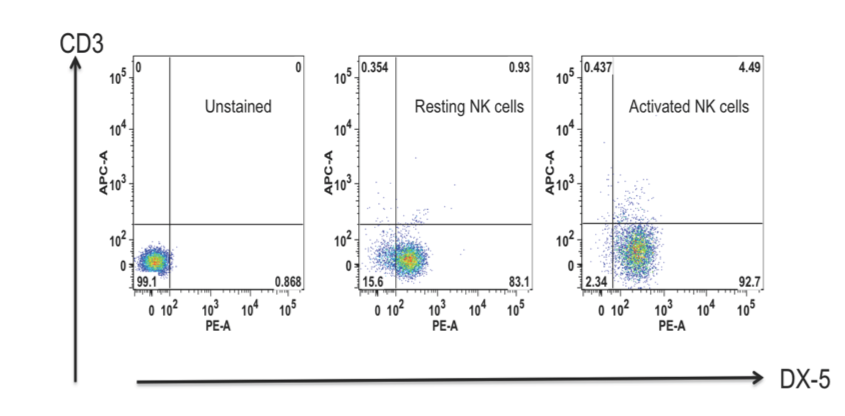
**

**Figure S3. Purity of the magnetic bead sorted NK cells used in the migration assays.** NK cells were isolated from spleen using the EasySep mouse NK negative selection kit as described in the Materials and Methods. Resting NK cells were used immediately after isolation. Activated NK cells were cultured with IL-2 (1000U/ml) for 4 days. Surface staining was used to measure the purity of NK cells using DX-5 PE and CD3 APC monoclonal Abs.

**S4**


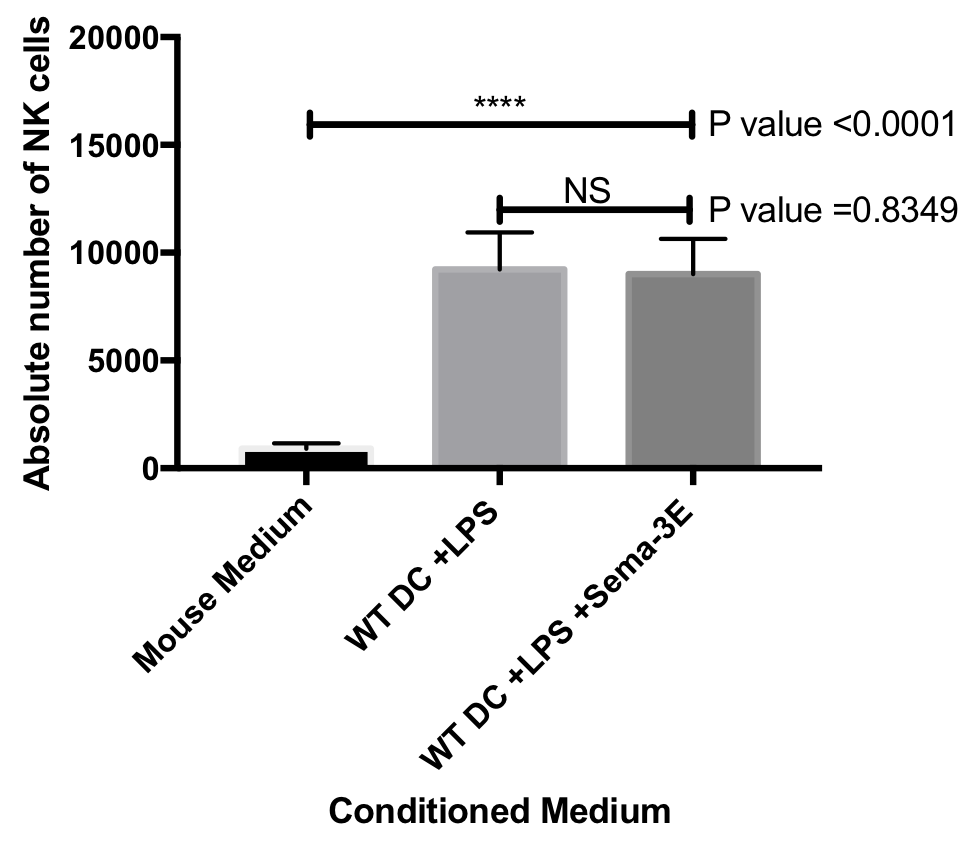


**Figure S4. Sema-3E has no effect on NK-cell migration towards conditioned medium from the LPS stimulated DC.** Migrations of the IL-2 activated NK cells towards Sema-3E^+/+^ LPS DC conditioned medium were examined in the trans-well migration assay. DCs were cultured in GM-CSF medium, DCs were further stimulated with LPS (1 μg/μl) for 12hours, and conditioned medium (D.C+LPS) was collected. Mouse medium was used as negative control. Recombinant Sema-3E (50 ng/ml) was added to the D.C+LPS conditioned medium at the time of running the migration assay. Statistical significance was established by One-way ANOVA was obtained to establish the comparison between three groups. Two-tailed student’s t-test were used to compare between two indicated groups at significance level of 0.05, (n=3 independent experiments). **** *p* value ≤ 0.0001, NS (non significant) *p* value > 0.05.

**S5**


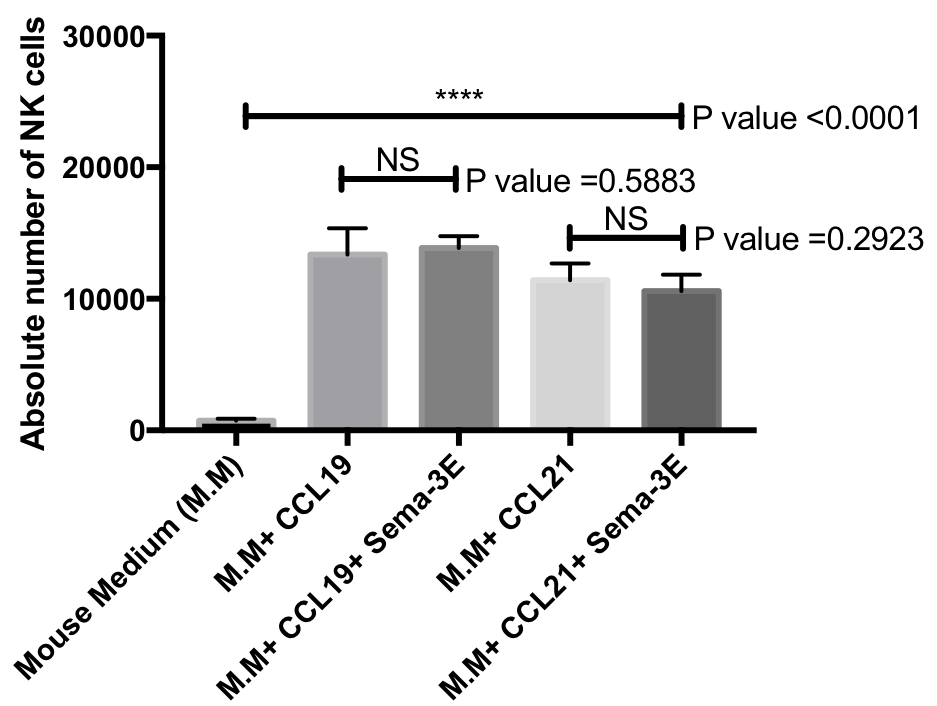


**Figure S5. Recombinant Sema-3E has no effect on NK-cell migration towards recombinant CCL19 or CCL21 chemokines.** NK cells were purified and activated with IL-2 for 4 days and NK cells migrations were assessed using trans-well migration assay. CCL19 chemokine (100 ng/ml) and CXCL21 chemokine (50 ng/ml) were added to mouse medium plus or minus recombinant Sema-3E. Mouse medium was used as a negative control where only CCL19 or CCL21 was used as a positive control. Statistical significance was established by One-way ANOVA was obtained to establish the comparison between three or more groups. Two-tailed student’s t-test were used to compare between two indicated groups at significance level of 0.05, (n=3 independent experiments). **** *p* value ≤ 0.0001, NS (non significant) *p* value > 0.05.

**S6**

**
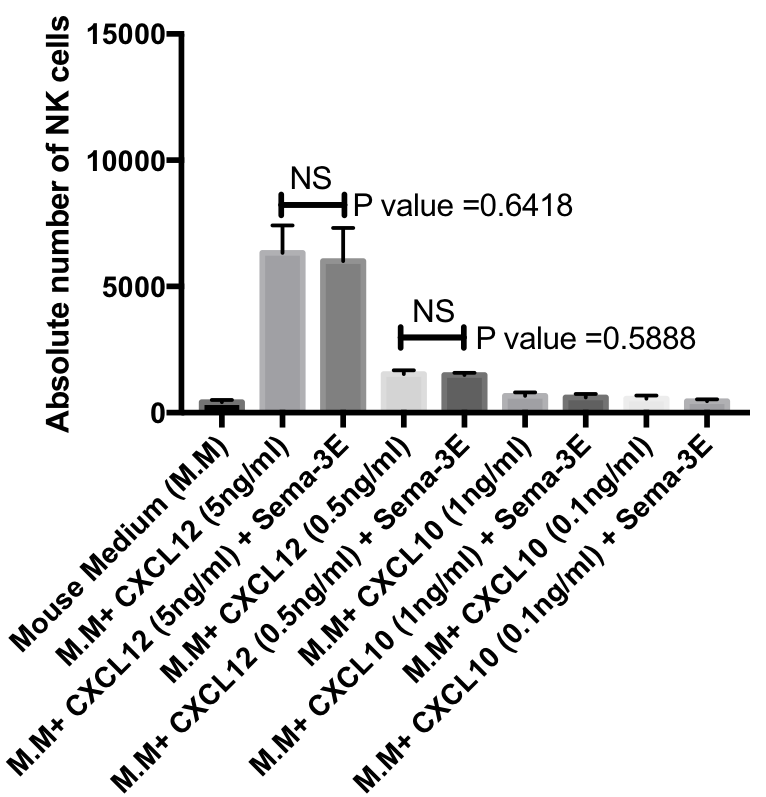
**

**Figure S6. Recombinant Sema-3E has no effect on NK-cell migration towards recombinant sub-optimal levels of recombinant CXCL10, CXCL12 chemokine.** Migrations of the IL-2 activated NK cells were measured by trans-well migration assay. CXCL12 chemokine (5 or 0.5 ng/ml) and CXCL10 chemokine (1 or 0.1 ng/ml) were added to mouse medium plus or minus recombinant Sema-3E at the time of running the migration assay. Mouse medium was used as a negative control where only CXCL12 or CXCL10 added to mouse medium was used as a positive control. Statistical significance was established by Two-tailed student’s t-test to compare between two indicated groups at significance level of 0.05, (n=3 independent experiments). NS (non significant) *p* value > 0.05.
